# Severe liver injury induced by minocycline in hepatitis B patient: a Case Report

**DOI:** 10.3389/fphar.2024.1516217

**Published:** 2024-12-19

**Authors:** Ran Wang, Yun Li, Xue Xia

**Affiliations:** Pharmacy Department Nanjing Lishui People’s Hospital, Zhongda Hospital Lishui Branch, Southeast University, Nanjing, Jiangsu Province, China

**Keywords:** minocycline, drug-induced liver injury, double plasma molecular adsorption system, chronic hepatitis B, immunomodulatory

## Abstract

Physical liver injury is an acute and potentially serious adverse event that may result in acute liver failure or even death. Diagnosis is often challenging. Minocycline, a semi-synthetic second-generation tetracycline, has high fat solubility and good tissue permeability. It is widely used for acne treatment. This report presents a 32-year-old female hepatitis B carrier who took minocycline (50 mg twice daily) for acne. After 1 week, she experienced fatigue, jaundice, abdominal bloating, and discomfort. Upon admission, laboratory tests revealed significantly elevated transaminase levels, ascites on abdominal ultrasonography, positive hepatitis B markers, impaired coagulation function, and a final diagnosis of subacute liver failure with chronic hepatitis B. Following discontinuation of minocycline and initiation of liver enzyme protection therapy, the patient’s liver and coagulation functions improved after undergoing artificial liver therapy combined with CRRT. This case highlights a rare occurrence of minocycline-induced liver injury in a hepatitis B carrier, emphasizing that acute liver injury is a potential and serious adverse effect of minocycline, particularly in patients with pre-existing liver disease.

## Introduction

Drug-induced liver injury (DILI) is a rare but potentially fatal adverse drug reaction. Epidemiological data based on the general population vary widely among countries, and the true incidence may be underestimated (Li et al., 2022). In China, the estimated annual incidence of DILI is at least 23.80/100,000, which is higher than in other countries and shows an increasing yearly trend (Li et al., 2022). DILI can be categorized into intrinsic, idiosyncratic, and indirect types based on pathogenesis, with each type having distinct clinical characteristics and associated drugs (Hoofnagle and Björnsson, 2019). Although most drug-induced liver injuries involve a specific mechanism, some drugs can cause damage through multiple mechanisms.

At least 1,000 drugs have been implicated in liver damage, with detailed information available on LiverTox (www.livertox.org) (Anonymous Author, 2012). Liver injury caused by tetracyclines is a serious adverse effect, with reports of severe cases in patients using tetracycline drugs (Zhan et al., 2019; Wang et al., 2023; Hamilton and Guarascio, 2019; Shi et al., 2021). Among these is minocycline, a semisynthetic tetracycline antibiotic. Minocycline exhibits both antibacterial and anti-inflammatory properties, making it a therapeutic option for dermatologic conditions such as acne and rosacea (Martins et al., 2021).

## Case presentation

A 32-year-old Chinese female presented with complaints of loss of appetite, fatigue, and yellowing of the skin and sclera. She had started taking minocycline capsules (50 mg twice daily) and vitamin B6 tablets (10 mg twice daily) for acne 1 week prior. During this period, she noticed yellow discoloration of her skin and urine, along with bloating. She denied using any other medications during this time. The patient reported being a healthy carrier of the hepatitis B virus, with normal liver and kidney function indicated in her annual physical exam. One month prior to admission, her liver and kidney function tests were as follows: AST 27.4 U/L, ALT 31.2 U/L, ALP 66 U/L, total bilirubin 8.2 µmol/L, urea 5 mmol/L, creatinine 77.4 µmol/L, and uric acid 187 mmol/L. She had no history of hypertension, diabetes, coronary artery disease, tuberculosis, surgical trauma, blood transfusion, or drug allergies.

Upon examination, she was afebrile (36.5°C), with a pulse rate of 104 beats/min and blood pressure of 118/78 mmHg. Her skin was dark yellow, and scleral icterus was present. No other abnormalities were detected.

Abdominal CT revealed fatty liver, cirrhosis, and possible liver nodules. B-ultrasound showed peritoneal effusion, gallbladder wall edema and thickening, and cirrhosis.

Laboratory results indicated significantly elevated AST (639 U/L), ALT (581 U/L), ALP (177 U/L), and total bilirubin (184.7 µmol/L, with direct bilirubin 86.8 µmol/L and indirect bilirubin 97.9 µmol/L). Albumin was 25.6 g/L. Coagulation tests revealed prolonged prothrombin time (28.9 s), PT-INR of 2.58, PTA of 22.3%, APTT of 49.5 s, fibrinogen of 0.75 g/L, and thrombin time of 28.9 s.

The patient was diagnosed with hepatitis B through hepatitis B testing. Results included a hepatitis B surface antigen quantification of 247.980 IU/L, surface antibody quantification of 0.000 mIU/L, e antigen quantification of 18.796 S/CO, e antibody quantification of 1.720 S/CO, and core antibody quantification of 8.000 S/CO. Tests for hepatitis A and hepatitis E were negative.

Immunoglobulin levels showed elevated IgG at 21.19 g/L and IgA at 4.66 g/L, with IgM at 1.96 g/L. Complement levels were low, with C3 at 0.13 g/L and C4 at 0.04 g/L. Results for other antibodies were all negative.

The thromboelastogram (TEG) results suggested that the patient’s fibrinogen function and platelet function were diminished.

During hospitalization, 16 reports showed the patient’s coagulation function had reached critical values. The patient underwent 12 blood transfusions, receiving a total of 2,350 mL of type A Rh-positive frozen fresh plasma and 57 IU of cryoprecipitate. Targeted transfusions and coagulation factor supplementation improved coagulation function.

The patient’s blood type was type A Rh-negative, complicating plasma matching for plasmapheresis. Consequently, the Double Plasma Molecular Adsorption System (DPMAS) combined with renal replacement therapy (RRT) was employed.

The patient received three DPMAS treatments on the third, fourth, and sixth days after admission. Throughout treatment, close monitoring of coagulation and liver function was maintained. Following these interventions, the patient’s coagulation function, liver function, and kidney function gradually returned to near-normal levels. The patient was discharged in stable condition.

## Discussion

Cases of minocycline-related adverse reactions have been reported previously (Wei et al., 2022; Shi et al., 2021; Yu et al., 2022). However, to our knowledge, this is the first documented case of minocycline-induced liver function impairment in a hepatitis B carrier. The patient, a female hepatitis B carrier, exhibited a slow onset and gradual progression of symptoms during minocycline use. Following the discontinuation of minocycline, symptomatic treatment was initiated, including liver function restoration, bilirubin reduction, and clotting correction. The patient’s ALT levels significantly decreased, transitioning from a hepatocellular injury pattern to a cholestatic injury pattern (Table 1). Although the current coagulation test indices remain above the upper limit, coagulation function has stabilized and improved significantly compared to levels at admission (Table 2). However, due to the pre-existing impaired liver function associated with hepatitis B, the patient’s bilirubin levels remain elevated (Table 3).

**TABLE 1:** Summary of liver function tests.

NO.	ALT (U/L)	AST (U/L)	ALP (U/L)	R
1st	307.00	272.00	94.00	8.2
2nd	151.00	108.00	48.00	7.9
3rd	83.00	53.00	40.00	5.2
4th	73.00	45.00	48.00	3.8
5th	58.00	49.00	52.00	2.8
6th	54.00	50.00	56.00	2.4
7th	63.00	110.00	80.00	2.0
8th	70.00	137.00	87.00	2.0
9th	65.00	127.00	77.00	2.1

ALT, alanine aminotransferase; AST, aspartate aminotransferase; ALP, alkaline phosphatase; ULN, upper limit of normal.

**TABLE 2:** Summary of coagulation function tests.

NO.	PT (sec)	PT-INR	PT% (%)	APTT (sec)	APTT_rati	Fbg (g/L)	TT (sec)
1st	28.90	2.58	22.30	49.50	1.74	0.75	28.90
2nd	46.30	4.20	13.10	>180.00	—	0.68	>150.00
3rd	>150.00	—	—	>180.00	—	0.50	55.30
5th	35.70	3.21	17.00	>180.00	—	1.48	49.30
6th	27.40	2.45	23.90	119.70	4.21	1.64	36.50
7th	>150.00	—	—	>180.00	—	1.20	>150.00
8th	22.80	2.02	30.30	53.20	1.87	1.42	21.40
9th	25.50	2.27	26.20	52.10	1.83	1.51	19.60
10th	30.30	2.71	22.40	121.00	4.26	1.23	42.00
11th	32.40	2.91	19.30	>180.00	—	1.19	>150.00
12th	21.10	1.87	33.40	50.30	1.77	1.80	22.10
13th	29.00	2.59	22.20	92.50	3.26	1.28	55.60
14th	21.40	1.90	32.80	52.70	1.86	1.28	20.90
15th	26.60	2.37	24.90	69.10	2.43	0.69	24.90
16th	25.40	2.26	26.40	81.00	2.85	0.61	26.80
17th	26.60	2.37	24.90	75.60	2.66	0.70	35.80
18th	29.00	2.59	22.20	79.70	2.81	0.68	36.72
19th	27.50	2.45	23.80	104.40	3.68	1.00	44.50
20th	23.70	2.11	30.50	65.80	2.32	0.88	28.80
21st	24.70	2.20	27.30	80.30	2.83	1.07	28.90

PT, prothrombin time; PT-INR, prothrombin time international normalized ratio; APTT, activated partial thromboplastin time; Fbg, Fibrinogen; TT, plasma thrombin time.

**TABLE 3:** Summary of serum bilirubin content.

NO.	Total bilirubin (µmol/L)	Direct bilirubin (µmol/L)	Indirect bilirubin (µmol/L)
1st	184.7	86.8	97.9
2nd	234.20	102.60	131.60
3rd	165.60	62.80	102.80
4th	143.00	51.50	91.50
5th	207.80	72.90	134.90
6th	169.70	67.80	101.90
7th	176.90	68.50	108.40
8th	170.30	80.60	89.70
9th	173.10	73.20	99.90
10th	181.50	76.60	104.90

Minocycline is a semi-synthetic second-generation tetracycline antibiotic with a broad antibacterial spectrum. Compared to other tetracyclines, it exhibits higher lipid solubility, improved tissue penetration, and a prolonged half-life. It is commonly used to treat skin and soft tissue infections, such as acne and periodontitis (Cunha et al., 2018). As the clinical use of minocycline has expanded, concerns regarding its safety have increased. Studies have reported a high incidence of adverse reactions to minocycline, estimated at 13.6% (Dominic, 2021), with liver function injury being one of the most commonly observed side effects. One study suggested that minocycline could induce hepatocyte damage and cholestatic liver injury (Wei et al., 2022). Alanine aminotransferase (ALT) is a sensitive biomarker for hepatocyte injury, especially when accompanied by elevated bilirubin levels. This pattern is a reliable indicator of liver injury in drug-induced liver injury (DILI) (Senior, 2016). Elevated alkaline phosphatase (ALP) levels typically indicate cholestatic damage, and when combined with increased gamma-glutamyl transferase (GGT), it suggests the ALP elevation is of hepatic origin. The ALT and ALP levels observed in this patient point to both hepatocellular and cholestatic liver damage. Research has shown that biliary excretion of drugs is a risk factor for hyperbilirubinemia. Many drugs that cause cholestasis or mixed DILI have been found to inhibit the bile salt export pump (BSEP) *in vitro*, which exacerbates liver toxicity, leading to cholestasis and bile acid retention (Sundaram and Bjornsson, 2017). Minocycline is primarily excreted via the biliary tract, which may contribute to its potential to cause cholestatic liver injury.

The exact risk factors for the development of drug-induced liver injury (DILI) are not yet fully understood. No significant difference in DILI incidence has been observed between genders. However, it has been noted that women are more prone to developing DILI with autoimmune features and are more likely to progress to acute liver failure once liver injury occurs (Suh, 2020). Chronic liver diseases and viral infections, such as hepatitis C virus (HCV) and human immunodeficiency virus (HIV), can lead to more severe forms of DILI. Immune reconstitution may be one of the mechanisms contributing to liver injury in these cases (EASLClinical Practice Guideline Panel: Chair:Panel membersEASL Governing Board representative:, 2019; Garcia-Cortes et al., 2020).

Minocycline is known to inhibit the proliferation of T lymphocytes (Szeto et al., 2010) and the chemotaxis of neutrophils (Garrido-Mesa et al., 2013). Garrido-Mesa et al. (2011) found that minocycline significantly suppresses the production of proinflammatory cytokines and chemokines, including IL-6, IL-8, IL-17, TNF-α, and IL-1β. The potential immunosuppressive effects of minocycline may contribute to the reactivation of hepatitis B virus (HBV) in patients with stable HBV infection. In such cases, the HBV genome persists in the liver even in patients with inactive or resolved HBV infection. The expression of these latent genomes is regulated by the immune system. Suppression or depletion of immune cells, particularly B cells, can lead to reactivation of what was thought to be a resolved HBV infection (Sarin et al., 2016), resulting in a hepatitis flare. HBV reactivation can cause serious, potentially fatal, hepatitis.

In this case, our patient was a woman who was a hepatitis B carrier, which may explain the development of her subacute liver failure. Most of the minocycline is excreted through bile, which could potentially disrupt the intestinal microbiome. The gut microbiota plays a crucial role in maintaining the body’s homeostasis and immune function. Emerging research suggests that disturbances in the microbiome and bacterial metabolism may increase the risk of DILI, although conclusive evidence remains lacking (Chen et al., 2021).

Diagnosing DILI is often challenging. There is no single clinical sign or laboratory value that can definitively confirm or exclude DILI. However, several emerging biomarkers, including glutamate dehydrogenase, keratin 18, glutathione S-transferase, sorbitol dehydrogenase, bile acids, cytochrome P450, and osteopontin, have been identified (EASLClinical Practice Guideline Panel: Chair:Panel membersEASL Governing Board representative:, 2019). These markers may enhance the specificity of DILI diagnosis and assist in prognosis. Currently, none of these biomarkers are routinely used in clinical practice, but they represent promising avenues for future research. At present, the diagnosis of DILI relies on a high index of suspicion. Once other potential causes of liver injury, such as viral infections, autoimmune diseases, liver malignancy, and vascular or metabolic disorders, have been excluded, and a temporal association with the administration of the suspected drug is observed, DILI can be diagnosed.

Our patient presented with a hepatocellular pattern of liver injury, which improved after the withdrawal of minocycline. A consistent temporal association was noted between minocycline intake and the development of liver damage. Vascular lesions were excluded through imaging, autoantibodies were tested negative, and other diseases with similar clinical presentations were ruled out. Additionally, the Roussel Uclaf Causality Assessment Method (RUCAM) score for this patient was 7, while the Naranjo Adverse Drug Reaction Probability Scale score was 6, classifying this case as “probable” DILI.

## Conclusion

This case report underscores the importance of caution in hepatitis B carriers regarding the use of drugs such as minocycline. Minocycline can not only cause severe liver damage but may also activate the hepatitis B virus, leading to hepatitis flare-ups. Clinicians who prescribe minocycline should be aware of the potential serious side effects associated with this commonly used drug. Drug-induced liver injury (DILI) is a diagnosis of exclusion, and prompt identification and discontinuation of the suspected drug are critical steps in managing these patients. Such actions can help improve prognosis and reduce mortality.

## Data Availability

The original contributions presented in the study are included in the article/supplementary material, further inquiries can be directed to the corresponding author.
